# Health-Related Behaviors and Odds of COVID-19 Hospitalization in a Military Population

**DOI:** 10.5888/pcd18.210222

**Published:** 2021-11-11

**Authors:** Bryant J. Webber, Michael A. Lang, David M. Stuever, James D. Escobar, Victoria F.H. Bylsma, Gregory G. Wolff

**Affiliations:** 1 Public Health and Preventive Medicine Department, US Air Force School of Aerospace Medicine, Wright-Patterson AFB, Ohio; 2 Department of Preventive Medicine and Biostatistics, Uniformed Services University of the Health Sciences, Bethesda, Maryland; 3 Eagle Integrated Services, LLC, Beavercreek, Ohio

## Abstract

**Introduction:**

Understanding the impact of behaviors on COVID-19 severity can improve health promotion strategies. We investigated the association between health-related behaviors and odds of hospitalization for COVID-19 in a cohort of military personnel.

**Methods:**

This case-controlled study compared all active-duty US Air Force service members hospitalized for COVID-19 between March 5, 2020, and March 10, 2021 (cases), with their geographically matched peers who had COVID-19 and were treated as outpatients (controls). We used logistic regression to compare cases and controls according to self-reported sleep duration, physical activity, dietary factors, binge alcohol consumption, and tobacco use — with and without adjustment for sociodemographic factors, body mass index, physical fitness level, pertinent disease history, and psychological distress — resulting in crude and adjusted odds ratios (ORs) with 95% CIs. The trend between sugar-sweetened beverage (SSB) consumption and hospitalization odds was assessed by using the Cochran-Armitage test.

**Results:**

Ninety-three hospitalized cases were matched to 372 ambulatory controls. Adjusting for baseline characteristics and other health-related behaviors, cases were more likely than controls to report fewer than 7 hours of sleep, compared with 7 to 9 hours (OR = 1.84; 95% CI, 1.07–3.16), and were more likely than controls to consume 3 or more SSBs per week, compared with fewer than 3 SSBs (OR = 1.74; 95% CI, 1.03–2.92). In a dose–response relationship, higher SSB consumption was associated with greater odds of being hospitalized (*P* value for trend = .02).

**Conclusion:**

Interventions that address short sleep duration and SSB consumption may reduce morbidity from COVID-19 among military service members and potentially in the broader US population.

**Summary**

**What is already known on this topic?**

Certain lifestyle-mediated diseases and health-related behaviors are associated with significant morbidity from COVID-19.

**What is added by this report?**

Among active-duty US Air Force service members, short sleep duration and sugar-sweetened beverage consumption were associated with higher odds of being hospitalized for COVID-19, even after controlling for sociodemographic factors, body mass index, physical fitness level, underlying disease history, and other health-related behaviors.

**What are the implications for public health practice?**

To mitigate the health effects of COVID-19, individuals should be encouraged to obtain adequate sleep and minimize sugar-sweetened beverage consumption.

## Introduction

Several chronic conditions have been implicated in the development of severe COVID-19 illness, including obesity (1,2), hypertension (2), diabetes mellitus (2), heart failure (2), malignancy (3), immunosuppression (4), and hypovitaminosis D (5). These conditions are causally or noncausally related to various health-related behaviors, some of which are independently associated with COVID-19 severity. For example, current and former smoking status is associated with disease progression (6), and physical inactivity increases risk of hospitalization, intensive care unit admission, and mortality (7,8).

Understanding the relationship between health-related behaviors and COVID-19 severity is important for guiding population-wide preventive strategies. We designed this study to determine whether short or long sleep duration, insufficient aerobic or strength-training activity, unhealthy dietary habits, binge alcohol consumption, tobacco use, and secondhand smoke exposure were associated with COVID-19 hospitalization in a military population. We chose this target population based on the potential national security ramifications of rampant illness, and the study affords a valuable opportunity for investigation due to availability of exposure and outcome data.

## Methods

This is a case-control study of active-duty US Air Force service members diagnosed with COVID-19 with a reported onset date between March 5, 2020, and March 10, 2021. Service members who were hospitalized for COVID-19 during the surveillance period served as cases. We defined controls as active-duty service members with COVID-19 who did not require hospitalization. (“Active duty,” as distinct from “reserve duty,” refers to members on full-time military status.) We randomly selected 4 controls for each case, matching on Air Force installation to account for potential geographic heterogeneity in virus variants and hospital admission criteria. All Air Force installations worldwide were eligible. Hospitalized cases and ambulatory controls were retrieved from the Disease Reporting System internet, the passive surveillance system for reportable medical events used by the US Department of Defense. Hospitalization status is a required variable in the system and is defined as admission to an inpatient ward; emergency department visits are ineligible. We included both confirmed and probable cases, which were defined according to the Department of Defense COVID-19 reporting case definition, adapted from the national definition then in use. Confirmed cases required a positive molecular amplification test, such as polymerase chain reaction. Probable cases required clinical compatibility and an epidemiologic link, or a positive antigen test, or a death certificate listing COVID-19 as either the underlying cause of death or a significant condition contributing to death (9). All reported cases were reviewed by the Air Force’s public health authority to validate the selected case classification. From the Disease Reporting System internet we also obtained age, sex, race or ethnicity, military rank, and date of onset (ie, the first day with signs or symptoms of COVID-19 or the day of diagnosis).

We retrieved anthropometric and physical fitness data from official tests archived in the Air Force Fitness Management System II. The annual Air Force fitness assessment includes measurements of height, weight, abdominal circumference, push-ups, sit-ups, and a 1.5-mile run. We calculated body mass index (BMI) as weight divided by height squared and stratified as normal (<25.0 kg/m^2^), overweight (25.0–29.9 kg/m^2^), and obese (≥30.0 kg/m^2^). The composite fitness score is calculated by summing the scores on each component: run time (up to 60 points), abdominal circumference (20 points), push-ups (10 points), and sit-ups (10 points). Per Air Force policy (10), scores on each component are adjusted for sex and age category. The fitness level is defined as excellent (composite score ≥90.0 and tested on all components), satisfactory (composite score 75.0–89.9 and tested on all components), pass (composite score ≥75.0 but did not test on all components), fail (composite score <75.0 or failure on any component), and exempt (did not test due to deployment, pregnancy, or a medical condition).

We retrieved self-reported disease history from the Tri-Service Periodic Health Assessment Questionnaire (PHAQ). Active-duty service members complete the online questionnaire annually, and answers are reviewed and signed by an authorized military health care provider (11). We defined a positive disease history as having a profile for the condition or as having been “bothered by” the condition in the 12 months preceding the PHAQ. We grouped diseases as cardiometabolic (hypertension, dyslipidemia, angina, abnormal heartbeat, congestive heart failure, and/or diabetes mellitus), respiratory (asthma, wheezing, and/or other lung problem), and immunodeficiency (malignancy and/or immune system dysfunction). These disease groups were independent, such that an individual could be classified as having all, any, or none. From the PHAQ we also assessed for the presence of psychological distress, defined as reporting a major life stressor within the prior month that caused significant concern or disturbed life, work, or relationships.

We also obtained self-reported health-related behaviors from the PHAQ. These questions are modeled after the Behavioral Risk Factor Surveillance System (12). We defined sleep duration as short (<7 hours per night), normal (7–9 hours per night), and long (>9 hours per night) over the 2 weeks before the PHAQ (13). We defined insufficient physical activity as failure to achieve 150 minutes per week of moderate-intensity activity, 75 minutes per week of vigorous-intensity activity, or the equivalent combination; and insufficient strength-training activity as fewer than 2 days/week (14). We defined inadequate vegetable and fruit consumption as fewer than 2 servings per day, respectively, over the prior 30 days (15). We defined sugar-sweetened beverage (SSB) consumption as 3 or more servings per week over the prior 30 days. SSBs are drinks sweetened with various forms of added sugars, and include regular soda, sweetened fruit drinks, sports or energy drinks, and sweetened coffee or tea. We defined binge drinking as consuming 6 or more alcoholic drinks on 1 or more occasions in the prior month. (Due to the questionnaire structure, we could not use the Substance Abuse and Mental Health Services Administration definition of 5 or more drinks for men or 4 or more drinks for women [16].) We stratified current tobacco use by type and defined it as using cigarettes, chewing tobacco, or electronic cigarettes in the prior 30 days. Finally, we defined secondhand smoking as reporting regular exposure to secondhand smoke.

Whenever possible, we used the fitness assessment and PHAQ immediately antecedent to the COVID-19 onset date, but not earlier than January 2018. For service members exempted from the antecedent fitness assessment, we used the last available assessment. In the absence of any prevenient values, we used the first values after the COVID-19 onset date. We considered variables missing if no records existed between January 1, 2018, and February 28, 2021. By using the Wilcoxon rank sum test, we compared cases and controls in terms of the time duration between their fitness assessment and PHAQ dates and their COVID-19 onset dates.

We categorized race or ethnicity as Hispanic, non-Hispanic Black, non-Hispanic White, non-Hispanic Asian, and other/unknown. This variable was included given known differences in COVID-19 hospitalization rates by race and ethnicity in the US (17) and the United Kingdom (18). We dichotomized military rank, a proxy of socioeconomic status, as enlisted or officer. Further stratification within enlisted and officer groups was unnecessary given strong correlation with age. We modeled age and BMI as both categorical and continuous variables and, for the latter, compared cases and controls by using the Wilcoxon rank sum test. All categorical baseline characteristics were compared by using the Wald χ^2^ test or Fisher exact test (when at least one cell had an expected value of ≤5).

For each health-related behavior (short and long sleep, physical inactivity, inadequate vegetable and fruit consumption, SSB consumption, binge alcohol consumption, tobacco use, and secondhand smoke exposure), we calculated descriptive statistics and a crude odds ratio (OR) with 95% CIs using the Wald χ^2^ test or Fisher exact test (when at least one cell had an expected value of ≤5). We calculated adjusted ORs with 95% CIs using multivariable logistic regression. Partially adjusted ORs incorporated age, sex, self-reported race or ethnicity, military rank, BMI, physical fitness level, disease history, and psychological distress, while fully adjusted ORs incorporated these baseline characteristics and all other health-related behaviors. This reflected a priori model considerations rather than a stepwise variable selection strategy.

We assessed the trend between dietary factors (vegetable, fruit, and SSB consumption) and hospitalization odds by using the exact Cochran-Armitage test. We used the 6 categories of the PHAQ: rarely, 1–2 servings/week, 3–6 servings/week, 1 serving/d, 2–3 servings/d, and ≥4 servings/d.

To assess the impact of missing race or ethnicity data and the use of the probable case definition, we conducted sensitivity analyses in which we calculated the fully adjusted ORs after excluding each set of individuals.

We used SAS version 9.4 (SAS Institute, Inc) to match controls with cases and to conduct all analyses. This project was reviewed by the Air Force Research Laboratory Institutional Review Board and determined to be a public health, nonresearch activity.

## Results

A total of 93 active-duty service members from 46 Air Force installations were hospitalized with COVID-19 during the surveillance period (confirmed n = 86; probable n = 7). These cases were matched to 372 ambulatory controls, service members from the same installations who were diagnosed with COVID-19 but did not require hospitalization (confirmed n = 346; probable n = 26).

Some service members had missing data for race or ethnicity (n = 129), BMI (n = 23), and physical fitness level (n = 15). (The discrepancy between BMI and fitness level was due to 8 members having the latter without an associated weight and height.) Among cases and controls, fitness assessments occurred by a respective median of 337 days and 377 days before the COVID-19 onset date (*P* = .03). Two fitness assessments (1 each for cases and controls) occurred after the onset date. Among cases and controls, PHAQs occurred by a respective median of 231 days and 203 days before the COVID-19 onset date (*P* = .32). Eleven PHAQs (1 case and 10 controls) occurred after the onset date.

Cases and controls were similar by age category, sex, race or ethnicity, military rank, BMI category, and disease history. Cases were more likely than controls to have a satisfactory physical fitness level, compared with an excellent level (crude OR = 1.90; 95% CI, 1.04–3.47), and they were less likely to report a major life stressor within the prior month that caused significant concern or disturbed life, work, or relationships (crude OR = 0.21; 95% CI, 0.05–0.87) (Table 1). Cases and controls had the same median age of 26 years (*P* = .92) and similar median BMI of 25.9 and 26.3 (*P* = .92).

**Table 1:** Characteristics of Active-Duty Air Force Service Members Hospitalized with COVID-19, Compared with Military Installation–Matched Ambulatory Controls With COVID-19, March 5, 2020, Through March 10, 2021 (N = 465)

Characteristic	Hospitalized Cases (n = 93)	Ambulatory Controls (n = 372)	Crude OR^a^ (95% CI)
	No. (%)		
**Age, y**			
<25	40 (43.0)	137 (36.8)	1 [Reference]
25–29	18 (19.4)	102 (27.4)	0.60 (0.33–1.12)
30–39	22 (23.7)	102 (27.4)	0.74 (0.41–1.32)
≥40	13 (14.0)	31 (8.3)	1.44 (0.69–3.00)
**Sex**			
Male	70 (75.3)	286 (76.9)	1 [Reference]
Female	23 (24.7)	86 (23.1)	1.09 (0.64–1.86)
**Race or ethnicity**			
Non-Hispanic White	42 (45.2)	189 (50.8)	1 [Reference]
Non-Hispanic Black	14 (15.1)	39 (10.5)	1.61 (0.81–3.24)
Non-Hispanic Asian	3 (3.2)	15 (4.0)	0.90 (0.16–3.39)^b^
Hispanic	3 (3.2)	6 (1.6)	2.24 (0.35–11.0)^b^
Other/missing	31 (33.3)	123 (33.1)	1.13 (0.68–1.90)
**Military rank**			
Enlisted	79 (85.0)	320 (86.0)	1 [Reference]
Officer	14 (15.0)	52 (14.0)	1.09 (0.58–2.07)
**Body mass index, kg/m^2^**			
<25.0	33 (35.5)	137 (36.8)	1 [Reference]
25.0–29.9	41 (44.1)	158 (42.5)	1.08 (0.65–1.80)
≥30.0	14 (15.1)	59 (15.9)	0.99 (0.49–1.98)
Missing	5 (5.4)	18 (4.8)	1.15 (0.31–3.54)^b^
**Physical fitness level^c^**			
Excellent	37 (40.0)	195 (52.4)	1 [Reference]
Satisfactory	22 (23.7)	61 (16.4)	1.90 (1.04–3.47)
Pass	23 (24.7)	78 (21.0)	1.55 (0.87–2.78)
Fail	1 (1.1)	6 (1.6)	0.88 (0.02–7.58)^b^
Exempt/missing	10 (10.8)	32 (8.6)	1.65 (0.75–3.64)
**Disease history**			
Cardiometabolic^d^	7 (7.5)	40 (10.8)	0.68 (0.29–1.56)
Respiratory^e^	3 (3.2)	22 (5.9)	0.53 (0.16–1.81)
Immunodeficiency^f^	0 (0.0)	4 (1.1)	0 (0–4.47)^b^
**Psychological distress^g^**	2 (2.2)	36 (9.7)	0.21 (0.05–0.87)

Abbreviation: OR, odds ratio.

a Comparing ambulatory controls to hospitalized cases for each variable.

b Based on Fisher exact test (all other odds ratios based on Wald χ^2^ test).

c According to the Air Force Fitness Assessment most proximally antecedent to the diagnosis, with few exceptions; the fitness level is based on a 1.5-mile timed run, push-ups, sit-ups, and abdominal circumference, with scores adjusted for age and sex.

d Self-reported history of hypertension, dyslipidemia, angina, abnormal heartbeat, congestive heart failure, or diabetes mellitus.

e Self-reported history of asthma, wheezing, or other lung problem.

f Self-reported history of malignancy or immune system dysfunction.

g Defined as reporting a major life stressor within the past month that caused significant concern or disturbed life, work, or relationships.

Self-reported health-related behaviors were similar between cases and controls with 2 notable exceptions. After adjusting for baseline characteristics and all other health-related behaviors, cases were more likely to report <7 hours of sleep per night, compared with 7–9 hours (fully adjusted OR = 1.84; 95% CI, 1.07–3.16). They were also more likely to consume 3 or more SSBs per week, compared with fewer than 3 SSBs (fully adjusted OR = 1.74; 95% CI, 1.03–2.92) (Table 2).

**Table 2:** Health-Related Behaviors of Active-Duty Air Force Service Members Hospitalized with COVID-19, Compared with Military Installation–Matched Ambulatory Controls With COVID-19, March 5, 2020, Through March 10, 2021 (N = 465)

Health-Related Behavior	Hospitalized Cases (n = 93), No. (%)	Ambulatory Controls (n = 372), No. (%)	Crude OR^a^ (95% CI)	Partially Adjusted OR^b^ (95% CI)	Fully Adjusted OR^c^ (95% CI)
**Sleep duration^d^**					
<7 h/night	59 (63.4)	205 (55.1)	1.46 (0.90–2.35)	1.84 (1.09–3.11)	1.84 (1.07–3.16)
>9 h/night	2 (2.2)	5 (1.3)	2.02 (0.18–13.0)^e^	1.93 (0.32–11.7)	1.98 (0.30–12.9)
**Physical inactivity**					
Insufficient aerobic^f^	13 (14.0)	46 (12.4)	1.15 (0.59–2.23)	1.01 (0.49–2.10)	1.06 (0.48–2.34)
Insufficient strength^g^	15 (16.1)	81 (21.8)	0.69 (0.38–1.27)	0.74 (0.39–1.40)	0.65 (0.32–1.30)
**Diet**					
Inadequate vegetable^h^	59 (63.4)	231 (62.1)	1.06 (0.66–1.70)	0.93 (0.56–1.55)	0.77 (0.41–1.47)
Inadequate fruit^h^	70 (75.3)	269 (72.3)	1.17 (0.69–1.97)	1.16 (0.67–2.02)	1.27 (0.64–2.50)
Sugar-sweetened beverage^i^	40 (43.0)	116 (31.2)	1.67 (1.05–2.65)	1.70 (1.04–2.80)	1.74 (1.03–2.92)
**Binge alcohol^j^**	1 (1.1)	4 (1.1)	1.00 (0.02–10.3)^e^	1.16 (0.11–12.0)	0.95 (0.07–12.3)
**Tobacco use^k^**					
Cigarettes	2 (2.2)	11 (3.0)	0.72 (0.08–3.39)^e^	0.69 (0.15–3.30)	0.40 (0.06–2.54)
Chewing tobacco	4 (4.3)	24 (6.5)	0.65 (0.22–1.93)	0.68 (0.22–2.04)	0.82 (0.25–2.67)
Electronic cigarettes	11 (11.8)	33 (8.9)	1.38 (0.67–2.84)	1.35 (0.62–2.94)	1.49 (0.60–3.67)
**Secondhand smoke^l^**	7 (7.5)	21 (5.6)	1.36 (0.56–3.30)	1.75 (0.67–4.57)	1.32 (0.45–3.88)

Abbreviation: OR, odds ratio.

a Comparing ambulatory controls to hospitalized cases for each variable.

b Comparing ambulatory controls to hospitalized cases for each variable, adjusting for baseline characteristics (age, sex, race or ethnicity, military rank, body mass index, fitness level, disease history, and psychological distress).

c Comparing ambulatory controls to hospitalized cases for each variable, adjusting for baseline characteristics (age, sex, race or ethnicity, military rank, body mass index, fitness level, disease history, and psychological distress) and the other health-related behaviors.

d Defined as average over the prior 2 weeks, compared with 7–9 h/night.

e Based on Fisher exact test (all other odds ratios based on Wald χ^2^ test).

f Defined as <150 min/wk of moderate-intensity activity, <75 min/wk of vigorous-intensity activity, or the equivalent combination.

g Defined as strength-training activity <2 d/wk.

h Defined as <2 servings/d.

i Defined as ≥3 servings/wk.

j Defined as ≥6 drinks on ≥1 occasion in the past month.

k Defined as using the product in the past 30 days.

l Defined as regular exposure to secondhand smoke.

SSB consumption followed a dose–response relationship, with greater consumption associated with greater odds of being hospitalized (*P* value for trend = .02) (Table 3). Dose–response was not statistically evident for consumption of vegetables (*P* = .38) or fruits (*P* = .31). Compared with individuals who reported sleeping 7–9 hours nightly and consuming fewer than 3 SSBs weekly, those who reported sleeping less than 7 hours nightly and consuming 3 or more SSBs weekly had even greater odds of hospitalization (crude OR = 2.31; 95% CI, 1.19–4.49; fully-adjusted OR = 3.09; 95% CI, 1.45–6.56).

**Table 3:** Sugar-Sweetened Beverage Consumption of Active-Duty Air Force Service Members Hospitalized with COVID-19, Compared With Military Installation–Matched Ambulatory Controls With COVID-19, March 5, 2020, Through March 10, 2021 (N = 465)

Consumption	Hospitalized Cases (n = 93), No. (%)	Ambulatory Controls (n = 372), No. (%)	Crude OR^a^ (95% CI)	Fully Adjusted OR^b^ (95% CI)	*P* Value^c^
Rarely	25 (26.9)	131 (35.2)	1 [Reference]	1 [Reference]	.02
1–2 Servings/wk	28 (30.1)	125 (33.6)	1.17 (0.65–2.12)	1.00 (0.52–1.93)	
3–6 Servings/wk	16 (17.2)	56 (15.1)	1.50 (0.74–3.02)	1.34 (0.61–2.92)	
1 Serving/d	15 (16.1)	34 (9.1)	2.31 (1.10–4.86)	2.27 (0.98–5.25)	
2–3 Servings/d	6 (6.5)	23 (6.2)	1.37 (0.51–3.70)	1.31 (0.44–3.87)	
≥4 Servings/d	3 (3.2)	3 (0.8)	5.16 (0.65–40.8)^d^	5.23 (0.67–40.9)	

Abbreviation: OR, odds ratio.

a Comparing ambulatory controls to hospitalized cases.

b Comparing ambulatory controls to hospitalized cases, adjusting for baseline characteristics (age, sex, race or ethnicity, military rank, body mass index, fitness level, disease history, and psychological distress) and the other health-related behaviors.

c *P* value for trend on 2-sided exact Cochran-Armitage test.

d Based on Fisher exact test (all other odds ratios based on Wald χ^2^ test).

The exclusion of individuals with missing race or ethnicity data or who were classified as probable cases did not substantially alter the findings beyond decreasing the precision of the effect estimates (Table 4).

**Table 4:** Sensitivity Analyses Associated With Health-Related Behaviors of Active-Duty Air Force Service Members Hospitalized With COVID-19, Compared With Military Installation–Matched Ambulatory Controls With COVID-19, March 5, 2020, Through March 10, 2021 (N = 465)

Health-Related Behavior	Hospitalized Cases (n = 93), No. (%)	Ambulatory Controls (n = 372), No. (%)	Fully Adjusted OR^a^ (95% CI)		
			Total Sample	Excluding 129 Individuals with Missing Race or Ethnicity Data	Excluding 33 Individuals Defined as Probable Cases
**Sleep duration^b^**					
<7 h/night	59 (63.4)	205 (55.1)	1.84 (1.07–3.16)	1.82 (0.94–3.53)	2.14 (1.20–3.81)
>9 h/night	2 (2.2)	5 (1.3)	1.98 (0.30–12.9)	0 (NA)	1.96 (0.30–13.0)
**Physical inactivity**					
Insufficient aerobic^c^	13 (14.0)	46 (12.4)	1.06 (0.48–2.34)	0.89 (0.33–2.37)	1.21 (0.54–2.74)
Insufficient strength^d^	15 (16.1)	81 (21.8)	0.65 (0.32–1.30)	0.79 (0.34–1.84)	0.56 (0.27–1.19)
**Diet**					
Inadequate vegetable^e^	59 (63.4)	231 (62.1)	0.77 (0.41–1.47)	0.95 (0.42–2.14)	0.75 (0.38–1.46)
Inadequate fruit^f^	70 (75.3)	269 (72.3)	1.27 (0.64–2.50)	1.69 (0.69–4.15)	1.12 (0.55–2.26)
Sugar-sweetened beverage^g^	40 (43.0)	116 (31.2)	1.74 (1.03–2.92)	1.47 (0.76–2.86)	1.79 (1.05–3.07)
**Binge alcohol^h^**	1 (1.1)	4 (1.1)	0.95 (0.07–12.3)	0 (NA)	1.01 (0.08–13.3)
**Tobacco use^i^**					
Cigarettes	2 (2.2)	11 (3.0)	0.40 (0.06–2.54)	0.35 (0.02–5.34)	0.37 (0.06–2.49)
Chewing tobacco	4 (4.3)	24 (6.5)	0.82 (0.25–2.67)	0.65 (0.12–3.48)	0.60 (0.16–2.32)
Electronic cigarettes	11 (11.8)	33 (8.9)	1.49 (0.60–3.67)	0.83 (0.22–3.20)	1.52 (0.57–4.04)
**Secondhand smoke^j^**	7 (7.5)	21 (5.6)	1.32 (0.45–3.88)	1.79 (0.42–7.71)	1.49 (0.48–4.61)

Abbreviations: NA, not applicable; OR, odds ratio.

a Comparing ambulatory controls to hospitalized cases for each variable, adjusting for baseline characteristics (age, sex, race or ethnicity, military rank, body mass index, fitness level, disease history, and psychological distress) and the other health-related behaviors.

b Defined as average over the prior 2 weeks, compared to 7–9 h/night.

c Defined as <150 min/wk of moderate-intensity activity, <75 min/wk of vigorous-intensity activity, or the equivalent combination.

d Defined as strength-training activity <2 d/wk.

e Defined as <2 servings/d.

f Defined as <2 servings/d.

g Defined as ≥3 servings/wk.

h Defined as ≥6 drinks on ≥1 occasion in the past month.

i Defined as using the product in the past 30 days.

j Defined as regular exposure to secondhand smoke.

## Discussion

We analyzed the association of health-related behaviors and COVID-19 severity, assessed by hospitalization, in a population of US Air Force service members. By matching on military installation and controlling for sociodemographic factors, BMI, physical fitness level, underlying disease history, and psychological distress, this study isolates the impact of health-related behaviors. Regardless of these baseline attributes, the odds of service members reporting less sleep and greater SSB consumption were higher in hospitalized cases when compared with nonhospitalized controls.

Obtaining 7 to 9 hours of sleep nightly is foundational for human health (13). Sleep regulates every bodily system affected by COVID-19 and reduces the risk of every major comorbidity associated with COVID-19 (13,19). In otherwise healthy adults, even moderate sleep deprivation blunts the immune response to influenza vaccination (20) and increases susceptibility to upper respiratory infection (21). This susceptibility may be mediated through or exacerbated by proinflammatory cytokines, which are secreted in higher concentrations after periods of even modest sleep restriction (22). Our study corroborates these findings. Holding other risk factors and health-related behaviors constant, service members with COVID-19 who averaged fewer than 7 hours of sleep, compared with their peers with COVID-19 who averaged 7–9 hours, had 84% greater odds of being hospitalized. The 57% overall prevalence of short sleep among cases and controls was higher than the 35% prevalence among US adults (23), suggesting that sleep is an important population health variable in Air Force service members. The relationship between long sleep and health outcomes warrants further investigation.

SSB consumption was associated with 74% increased odds of hospitalization when dichotomized at 3 or more servings per week versus fewer than 3 servings per week, and a dose–response relationship was evident when assessed as a trend of ordinal categories. These findings are striking given the low prevalence of SSB intake among cases and controls. Compared with the 63% of US adults who report consuming SSBs at least daily (24), only 18% of service members in this study reported a similar intake. We are not aware of any literature assessing the impact of SSB consumption on COVID-19 severity; however, many studies have reported a relationship between hyperglycemia, with or without overt diabetes mellitus, and severe COVID-19 (25–27). The pathogenic mechanism is not entirely clear but likely relates to a hyperglycemia-induced pro-inflammatory milieu. In vitro studies demonstrate a direct correlation between glucose levels and both viral replication and cytokine expression within infected monocytes (28,29) and a positive association between glucose levels and gene expression of IL-1β and IL-12 (30). Given these observational studies, the biochemical plausibility, and the lack of harm associated with this recommendation, we encourage local and national strategies that promote healthier alternatives to SSB consumption across the population.

The reason why service members who reported a significant life stressor had lower crude odds of hospitalization for COVID-19 is unclear. We anticipated the opposite finding because acute stress has been linked to impaired immune function and higher risk of respiratory infections (31). A potential explanation is confounding by indication, such that service members with greater self-awareness or greater psychosocial resilience were more likely to report life stressors. In other words, we speculate that openness to report stressors is more indicative of response to SARS-CoV-2 infection than the presence or absence of such stressors. The overall prevalence of psychological distress among cases and controls was 8%. Although this prevalence is lower than the 13% of US adults in a recent survey (32), strategies are warranted to increase self-awareness, to decrease stigma associated with care-seeking, and to link distressed service members with appropriate base and community resources.

The null findings in this study are also interesting. Contrary to our expectations, inadequate consumption of vegetables and fruits, physical inactivity, and tobacco use were not associated with hospitalization in crude and adjusted models, and trends for vegetable and fruit consumption were not significant. These findings may be due to the small sample size of hospitalized cases in this study. The null finding for physical inactivity may reflect its low prevalence in this cohort of active-duty service members. Whereas more than 75% of American adults fail to achieve the minimum recommended level of aerobic and strength-training activity (14), fewer than 30% of individuals in this study fell short of the guideline recommendation. This finding is not surprising as Air Force service members are required to meet a minimum level of physical fitness (10). Although this study focused on health-related behaviors, it should be noted that members who received a “satisfactory” score on their fitness assessment had 90% higher crude odds of hospitalization compared with their peers who received an “excellent” score. Rather than discounting the value of nutrition, physical activity, and tobacco avoidance, this study underscores the importance of factors that achieved significance despite limited power.

In addition to the small sample size, this study has 6 important limitations. First, data were self-reported and some questions considered a condensed timeframe. Questions related to major life stressors and sleep duration, for example, were restricted to 30- and 14-day windows before the PHAQ, respectively. Self-reported surveys also raise the issues of recall and reporting biases. Exposure misclassification may be present due to perceived stigma and military career consequences, especially concerning alcohol and tobacco use, as these questionnaires are reviewed by military medical personnel. We suspect any misclassification would be nondifferential by outcome, thus biasing results toward the null. Second, missing baseline data were present for some variables, most notably for race or ethnicity, which has been associated with COVID-19 severity (17,18); however, the sensitivity analysis disputes extensive bias. Third, hospitalization status may have been undercounted. If service members were diagnosed with COVID-19, reported in the system, and later hospitalized, the hospitalization status may not have been updated by the reporting public health unit. Fourth, vaccination information was unavailable. Because mRNA vaccines appear to be more effective at preventing COVID-19–related hospitalization than COVID-19 infection (33), and because this vaccination was optional for military members during the surveillance period (34), vaccination status may be a confounding variable in this analysis. This confounding would be particularly problematic if vaccine uptake was differential by health-related behaviors, in particular if individuals who obtained less sleep or who consumed greater quantities of SSBs were also less likely to receive the vaccine. Fifth, for a small percentage of cases and controls, fitness assessment and PHAQ data were collected after the COVID-19 onset date, which could introduce bias associated with reverse temporality (eg, if infection impaired performance on the physical fitness assessment or prompted changes in health-related behaviors).

Finally, hospitalization is an imperfect surrogate for severity. Nondisease considerations such as improvised isolation and psychosocial issues can affect decisions to hospitalize a patient. However, we believe these factors would be rare for active-duty military service members. Hospitalization criteria should be similar across Air Force military treatment facilities, and potential heterogeneity was accounted for by geographical matching of cases and controls. Furthermore, the use of hospitalization as an indicator of COVID-19 severity is widespread in studies of civilian populations (1,2,6–8).

Because this study was conducted in a military context, external validity to civilian populations may be questioned. For example, the findings may not apply to children and older adults, or to young and middle-aged adults who are ineligible for military service due to medical or educational reasons. Conversely, the findings may not apply to all members of the US Armed Forces given heterogeneity in physical fitness and health requirements among the military services and among units within each service (eg, US Air Force Special Warfare operators and other US Air Force career fields). The inclusion of cases detected outside the US may be considered another threat to external validity, but given the rapid international spread of variant strains, this may be a strength of the study, especially since controls were matched on military installation.

With SARS-CoV-2 variants spreading, actionable public health messages are needed to empower individuals and communities. Adequate sleep and reduced consumption of SSBs, like vaccination and respiratory etiquette, are controllable behaviors that could mitigate hospitalization associated with COVID-19. Although causality cannot be presumed and generalizability beyond the military should proceed cautiously, these behaviors appeared to be protective irrespective of BMI, baseline physical fitness, and disease history. Prioritizing sleep and limiting SSB consumption are evidence-based messages that may reduce susceptibility to severe COVID-19 illness among active-duty military personnel.
